# Etiology of Hydronephrosis in adults and children: Ultrasonographic Assessment in 233 patients

**DOI:** 10.12669/pjms.37.5.3951

**Published:** 2021

**Authors:** Sultan Abdulwadoud Alshoabi, Dahhan Saleh Alhamodi, Mohammed Ali Alhammadi, Abdullah Fahad Alshamrani

**Affiliations:** 1 Sultan Abdulwadoud Alshoabi, Department of Diagnostic Radiology Technology, College of Applied Medical Sciences, Taibah University, Almadinah Almunawarah, Kingdom of Saudi Arabia; 2 Dahhan Saleh Alhamodi, Unit of Ultrasound Imaging, Department of Radiology, Amran Hospital, Amran, Republic of Yemen; 3 Mohammed Ali Alhammadi, Department of Radiology and Medical Imaging, Prince Mohamad bin Abdulaziz Hospital, National Guard, Almadinah Almunawarah, Kingdom of Saudi Arabia; 4 Abdullah Fahad Alshamrani, Department of Diagnostic Radiology Technology, College of Applied Medical Sciences, Taibah University, Almadinah Almunawarah, Kingdom of Saudi Arabia

**Keywords:** Etiology, Hydronephrosis, Calculi, Pregnancy-induced hydronephrosis, Ultrasound imaging

## Abstract

**Objectives:**

Hydronephrosis (HN) is dilatation of the collecting system of the kidney due to obstruction of urine outflow. This study intended firstly, to investigate the efficacy of ultrasound (US) imaging to determine the cause of HN, and secondly, to list the causes of HN.

**Methods:**

In this retrospective study, 233 patients with HN were scanned to determine the cause of the HN in the period from 1^st^ January 2016 to 31^st^ October 2017. Categorical results were written as frequencies and percentages.

**Results:**

Out of 233, 91.41% were adults and 8.58% were children (P<0.001), 66.10% were male and 33.90% were female (P<0.001). In 55.36%, HN was in the right kidney and 44.64% was in the left (P=0.116). Exactly 58% of patients were suffering from grade-2, 21.5% grade-3, 11.6% grade-1, and 8.2% grade-4 HN. US imaging can determine the cause of HN in 70.4% of patients. Kidney or ureteric calculi were the cause of HN in 54.1% of cases, reflux was in 7.3%, and pelviureteric junction (PUJ) stenosis was in 3.9%.

In cases of calculi induced HN, 25.3% of the calculi were in the vesicoureteric (VUJ) junction, 21.5% were in the renal pelvis, 6.4% were in the PUJ or upper ureter, and only 0.9% were in the middle ureter.

**Conclusion:**

Ultrasound imaging can determine the cause of HN in more than two thirds of patients. Calculi are the most common cause of HN even in children and are most common in the VUJ junction.

**Abbreviations:**
HN: Hydronephrosis,
US: Ultrasound,
PUJ: Pelviureteric Junction,
VUJ: Vesicoureteric Junction,
SFU: Society of Fetal Urology,
MHz: Megahertz,
SPSS: Statistical Package for the Social sciences,
IBM: International Business Machines,
NY: New York,
CI: confidence interval.

## INTRODUCTION

Hydronephrosis (HN) is dilatation of the renal collecting system of the kidney due to obstruction of urine outflow in any part of the urinary tract. It can present solely or together with dilatation of the ureter in an entity called hydroureternephrosis. HN can present as unilateral or bilateral, acute or chronic at any age.^1^

According to the Society of Fetal Urology (SFU) classification system, HN is classified into four grades; Grade-1; dilatation of the renal pelvis only. Grade-2; dilatation of the renal pelvis and major calyces. Grade-3; dilatation of the renal pelvis and major and minor calyces. Grade-4; dilatation of the renal pelvis and all calyces with thinning of the renal parenchyma. The SFU classification system is used also in adults.^2^ This classification system has good intra observer and interobserver reliability and is recommended for assessment of neonatal HN.^3^

The most common reported causes of HN are kidney or ureteric calculi, pregnancy, pelviureteric junction (PUJ) stenosis or bladder outlet obstruction.^4^ Ultrasound (US) imaging is commonly used, universally available, non-invasive and radiation free imaging modality. It is highly valuable in diagnosing and grading of HN.^5^

This study was designed to investigate the efficacy of US in determining the cause of HN which is a common medical problem in all age groups and the detection of the cause of HN is a critical point for planning of the management. Moreover, the study was carried out to examine the causes of HN in adults and children due to lack of enough similar previous studies about causes of HN in adults and children which is a common and important topic and many previous studies were concentrated only on prenatal and neonatal HN. This work will be beneficial for ultrasonographists, radiologists, urologists and emergency practitioners who are usually interested in detecting the cause of HN as a frequent and important problem corresponds to their daily work.

This study also elucidate the distribution of HN in the right and left kidneys and in male and female gender and explained the cause of predilection of HN to right side in female.

## METHODS

This cross-sectional retrospective study involved 233 patients who underwent abdominal US imaging and diagnosed with HN at the US unit of Amran hospital in Yemen during the period from 1^st^ January 2016 to 31^st^ October 2017. This study involved both adults and children. Exclusion criteria include prenatal HN because a lot of previous studies satisfy this topic.

### Procedure

All patients were scanned by the same radiologist who has eight years of experience in abdominal ultrasonography. A 3.5 MHz curved transducer of Medison, Sono ex-model six color Doppler machine was used in scanning of all patients. Each patient was scanned in supine and oblique positions to demonstrate and grading HN. Each kidney was examined in longitudinal and transverse sections with an examination of the whole ureter and urinary bladder to demonstrate the cause of HN.

Patients were scanned by the same highly experienced radiologist following the protocol of ultrasound imaging of the kidneys.

### Statistical Analysis

The collected data were analyzed using the statistical package for social sciences (SPSS), IMB, version 23 for windows (Armonk, NY: IBM Corp. 2015). Chisquare test was used to compare urinary calculi in gender and side. P value was assumed to be significant when <0.05. Cross tabulation was used to analyze relationship between gender and side distribution of urinary calculi. Results were reported as frequencies and percentages in categorical data.

### Ethical Approval

Institutional ethical approval was obtained for this study. Confidentiality of the patients was assured during and after this study.

## RESULTS

This study involved 233 patients who underwent abdominal US imaging and diagnosed as HN. 91.41% were adults and 8.58% were children, 66.10% were male and 33.90% were female. HN was unilateral in 88.85% and bilateral in 11.5%. HN was in the right kidney in 55.36% and 44.64% was in the left (Table-I).

**Table-I:** Age, sex and side groups of hydronephrosis.

Variables	Categories	Numbers	Percentage	P-value
Age	Adult	213	91.41	<0.001
	Child	20	8.58	
	Total	233	100%	
Sex	Male	154	66.10	<0.001
	Female	79	33.90	
	Total	233	100%	
Kidney	Unilateral	207	88.85	<0.001
	Bilateral	26	11.15	
	Total	233	100%	
Side	Right	129	55.36	0.116
	Left	104	44.64	
	Total	233	100%	

Table shows significance association between hydronephrosis with adults and male gender (P<0.001), but no significant association between hydronephrosis and any side (P=0.116).

**Fig.1:**
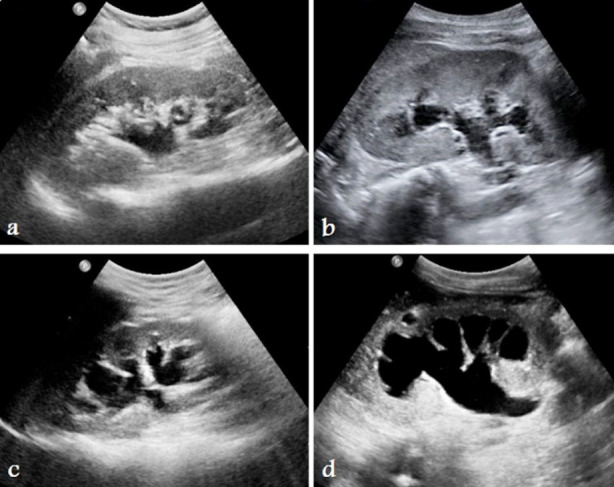
Ultrasound images of right kidney of different patients shows a) grade-1, b) grade-2, c) grade-3, and d) grade-4 hydronephrosis according to SFU grading system.

Table-II shows that 58% of patients were suffering from mild (Grade-2) HN, 21.5% moderate (Grade-3), 11.6% minimal (Grade-1), and 8.2% severe (Grade-4) HN (Fig.1).

**Table-II:** Grades of hydronephrosis.

Categories	Numbers	Percentage
Grade-1	27	11.6
Grade-2	137	58.8
Grade-3	50	21.5
Grade-4	19	8.2

Total	233	100

Table reveals that grade-2 hydronephrosis was the most common then grade-3.

Grade- 1(Mild): Dilatation of the renal pelvis only.

Grade- 2(Mild): Grade-1+ dilatation of major calyces.

Grade- 3(Moderate): Grade-2+ dilatation of all calyces.

Grade- 4(Severe): Grade-3+ thinning of the renal parenchyma.

Ultrasound imaging can determine the cause of HN in 70.4% of patients with HN. Kidney or ureteric calculi was the cause of HN in 54.1% of cases, 7.3% of cases were reflux HN, PUJ stenosis was the cause in 3.9%, and residual HN was determined in 1.7% of patients (Table-III). Regarding causes in children, calculi were the cause in 30% of cases, PUJ stenosis in 20%, reflux HN in 15%, residual in 5%, and 25% with no determined cause (Table-IV).

**Table-III:** Causes of hydronephrosis in all patients.

Categories	Numbers	Percentage
Calculi	126	54.1
Reflux	17	7.3
PUJ stenosis	9	3.9
Pregnancy	8	3.4
Residual	4	1.7
Not determined	69	29.6

Total	233	100

Table reveals that ultrasound can detect the cause of hydronephrosis in 164 (70.38%) of cases of hydronephrosis and calculi were the cause in 126 (54.10%) of patients.

**Table-IV:** Causes of hydronephrosis in children.

Categories	Numbers	Percentage
Calculi	6	30
PUJ stenosis	4	20
Reflux	3	15
Residual	1	5
Not determined	5	25

Total	20	100

Table reveals that ultrasound can detect the cause of hydronephrosis in 164 (70.38%) of cases of hydronephrosis and calculi were the cause in 126 (54.10%) of patients.

In cases of calculi induced HN, 25.3% of calculi were in the vesicoureteric junction (VUJ) or distal part of ureter, 21.5% were in the renal pelvis, 6.4% were in the PUJ or upper ureter, and only 0.9% were in the middle ureteric part (Table-V).

**Table-V:** Common sites of detected calculi in cases of hydronephrosis.

Categories	Numbers	Percentage
Renal pelvis	50	21.5
Upper ureter	15	6.4
Mid ureter	2	.9
Lower ureter	59	25.3
Other causes	38	16.3
Not determined	69	29.6

Total	233	100

Table reveals that calculi were common in distal part of ureter, then renal pelvis, followed by upper ureter.

The cross-tabulation test between sex and side of HN shows predilection of HN to the right side only in female but not statistically significant (p=0.072), (Odds ratio= 0.832), (95% confidence interval (CI) 0.666-1.054), (Table-VI).

**Table-VI:** Cross-tabulation test between sex and side of hydronephrosis.

Categories	Right	Left	
Male	80 (51.9%)	74 (48.1%)	154
Female	49 (62%)	30 (38%)	79

Total	129 (55.4%)	104 (44.6%)	233

Table reveals slight predilection of hydronephrosis to the right side only in female but not statistically significant (p=0.072).

## DISCUSSION

HN is a widespread health problem worldwide that can be present at any age because of multiple causes. Determining the cause of HN is an essential when planning the treatment.

In a previous study by Riddell et al., the sensitivity of bedside US imaging to detect unilateral HN was 72-83%.^6^ In this study, we found that US imaging can determine the cause in 70.4% of all patients with HN. Another previous study by Moş et al. reported that transabdominal US imaging can identify HN in 88.94% and can identify ureteric calculi in nearly 73% of patients.^7^ Sternberg et al. reported that HN on US imaging has a 77% positive predictive value for diagnosis of ureteric calcui with 71% negative predictive value.^8^

In this study, we found that HN was more common in male than in female, mild (grade-2) HN was the most common and ureteric or kidney calculi were the most common cause even in children. These results were consistent with that of Nuraji and Hyseni., who reported that HN was more common in grade-2 and in males with kidney and ureteric stones were the most common cause.^4^ These results are also consistent with a previous study by Abu-Ghazzeh and Abdu-Alro’f, who reported that calculi were the most common cause of obstructive HN and were most common in the VUJ.^9^

Another similar study by Hansen et al. reported that calculi were the most common cause of HN in adults. He reported that renal pelvic, PUJ and VUJ calculi can be detected by US. However, calculi in the ureter are difficult to be detected due to obscuration by bowel gases.^10^ This explains the low detection rate of ureteric calcui by US which reported in the results of this study. Calculi are the cause of HN in 54.1% of patients in the current study. This is consistent with another previous study by Alshoabi, who reported that calculi were the cause of HN in 60% of cases.^11^

This study showed predilection of HN to male gender. This predilection was explained by the results of a previous study by Ahmed et al. who reported that urinary tract calculi, which are the most common cause of HN, are formed more common in males due to hormonal effects.^12^

The high prevalence of nephrolithiasis in males is attributed to the effect of sex hormones such as androgens on some lithogenic risk factors which increases excretion and deposition of calcium oxalate in the pelvicalyceal system and kidney stone formation. In addition, estrogen decreases excretion of urinary oxalate and contribute in nephrolithiasis.^12^

The current study revealed predilection of HN to the right side in female gender. This can be explained by that pregnancy-induced HN was the cause in 3.4% of cases and was reported more common on the right side.^13^

## CONCLUSION

Ultrasound imaging is an effective method to diagnose hydronephrosis which is a very common medical problem predominantly in adults, more in male, and slightly predominate on the right side. Ultrasound imaging can determine the cause of hydronephrosis in more than two thirds of patients. Ureteric or kidney calculi were the most common cause of hydronephrosis followed by reflux. Pelviureteric junction stenosis and residual hydronephrosis were uncommon causes. Calculi were determined in vesicoureteric junction, then in the renal pelvis and upper ureteric part and rarely in the middle ureteric part.

### Significant statement

This study focused on studying the ability of US imaging to determine the cause of HN which is a common medical problem worldwide. US is a widely available and safe medical imaging modality that can frequently determine the cause of HN and calculi are the most common determined cause. This data is highly valuable to ultrasonographists, radiologists, urologists and physicians who commonly using ultrasound imaging.

### Authors’ Contribution:

**SAA** Prepared the manuscript and critically reviewed and approved the final draft and is responsible for the accuracy of the work.

**DSA** collected and organized data.

**MAA** interpreted data.

**AFA** revised and final approval of the manuscript.
